# SDS-Stabilized CuInSe_2_/ZnS Multinanocomposites Prepared by Mechanochemical Synthesis for Advanced Biomedical Application

**DOI:** 10.3390/nano11010069

**Published:** 2020-12-30

**Authors:** Erika Dutková, Zdenka Lukáčová Bujňáková, Oleh Sphotyuk, Jana Jakubíková, Danka Cholujová, Viera Šišková, Nina Daneu, Matej Baláž, Jaroslav Kováč, Jaroslav Kováč, Jaroslav Briančin, Pavlo Demchenko

**Affiliations:** 1 Department of Mechanochemistry, Institute of Geotechnics, Slovak Academy of Sciences, Watsonova 45, 04001 Košice, Slovakia; bujnakova@saske.sk (Z.L.B.); balazm@saske.sk (M.B.); briancin@saske.sk (J.B.); 2 Faculty of Science and Technology, Jan Dlugosz University, Al. Armii Krajowej, 13/15, 42201 Czestochowa, Poland; olehshpotyuk@yahoo.com; 3 Department of Optical Glass and Ceramics, Vlokh Institute of Physical Optics, 23, Dragomanov Str., 79005 Lviv, Ukraine; 4 Biomedical Research Center, Cancer Research Institute, Slovak Academy of Sciences, Dúbravská cesta 9, 84505 Bratislava, Slovakia; Jana.Jakubikova@savba.sk (J.J.); exondach@savba.sk (D.C.); exonvisi@savba.sk (V.Š.); 5 Centre of Advanced Material Application, Slovak Academy of Sciences, Dúbravská cesta 5807/9, 84511 Bratislava, Slovakia; 6 Advanced Materials Department, Jozef Štefan Institute, Jamova 39, 1000 Ljubljana, Slovenia; nina.daneu@ijs.si; 7 Institute of Electronics and Photonics, Slovak University of Technology, 81219 Bratislava, Slovakia; jaroslav.kovac@stuba.sk (J.K.); jaroslav_kovac@stuba.sk (J.K.J.); 8 Department of Inorganic Chemistry, Ivan Franko National University of Lviv, 107, Tarnavskogo Str., 79017 Lviv, Ukraine; pavlo.demchenko@lnu.edu.ua

**Keywords:** mechanochemical synthesis, CuInSe_2_/ZnS, sodium dodecyl sulphate (SDS), nanocrystals, microstructure, surface properties, optical properties, biological properties

## Abstract

The CuInSe_2_/ZnS multiparticulate nanocomposites were first synthesized employing two-step mechanochemical synthesis. In the first step, tetragonal CuInSe_2_ crystals prepared from copper, indium and selenium precursors were co-milled with zinc acetate dihydrate and sodium sulfide nonahydrate as precursors for ZnS in different molar ratios by mechanochemical route in a planetary mill. In the second step, the prepared CuInSe_2_/ZnS nanocrystals were further milled in a circulation mill in sodium dodecyl sulphate (SDS) solution (0.5 wt.%) to stabilize the synthesized nanoparticles. The sodium dodecyl sulphate capped CuInSe_2_/ZnS 5:0-SDS nanosuspension was shown to be stable for 20 weeks, whereas the CuInSe_2_/ZnS 4:1-SDS one was stable for about 11 weeks. After sodium dodecyl sulphate capping, unimodal particle size distribution was obtained with particle size medians approaching, respectively, 123 nm and 188 nm for CuInSe_2_/ZnS 5:0-SDS and CuInSe_2_/ZnS 4:1-SDS nanocomposites. Successful stabilization of the prepared nanosuspensions due to sodium dodecyl sulphate covering the surface of the nanocomposite particles was confirmed by zeta potential measurements. The prepared CuInSe_2_/ZnS 5:0-SDS and CuInSe_2_/ZnS 4:1-SDS nanosuspensions possessed anti-myeloma sensitizing potential assessed by significantly reduced viability of multiple myeloma cell lines, with efficient fluorescence inside viable cells and higher cytotoxic efficacy in CuInSe_2_/ZnS 4:1-SDS nanosuspension.

## 1. Introduction

Multiparticulate nanocomposites (multinanocomposites (MNC)) represents a group of phase-distinguished nanostructured substances possessing wide application in biomedicine as advanced media utilizing unique exploitation properties inaccessible for their unicomponent precursors [1]. Nowadays, rich family of such materials can be well exemplified by MNC containing ZnS nanoparticles complemented with other chalcogenide compounds [2,3,4,5], like semiconductor CuInSe_2_ [3], ensuring unified functionality of a whole nanosystem with improved optical properties due to elimination of surface non-radiative recombination defects. So biparticulate CuInSe_2_/ZnS MNC with equal ratio between components exactly reaching 1:1 are perspective highly-luminescent “green” materials for bioimaging free of hazardous additives [6,7,8,9]. Recently, several techniques have been successfully developed to synthesize inorganic-coated CuInSe_2_ nanomaterials, such as electron beam evaporation [10], large-scale synthesis using gelatin and thioglycolic acid as dual stabilizers in electric pressure cooker [11] and organic phase high temperature route combined with alloying [12]. Stable equi-component CuInSe_2_/ZnS colloidal nanocrystals have been prepared employing different nanostructurization technologies as described in more details elsewhere [7,8,13,14].

From technologically-guided and bio-applicability tuning challenges, the most promising approach seems to have highly-stabilized biparticulate CuInSe_2_/ZnS MNC with variable ratio between components, like it is achieved in other ZnS-based prototypes [15,16,17]. To improve bioimaging ability and cytotoxicity of these MNC, stable nanosuspensions should be prepared avoiding parasitic inter-particulate aggregation and agglomeration processes. Sodium dodecyl sulphate (SDS) is known to be one of the best biocompatible moderately toxic anionic surfactants for this purpose, widely used in pharmaceutical and industrial (building, chemical, detergency and textile) applications [18,19]. Its potential toxicity is also a subject to research [20,21]. At the final stage, the top-down approach employing high-energy mechanochemical synthesis in wet stirred media can be used to produce nanosuspensions, as it was noted in a number of recent publications [15,17,22,23,24,25,26].

To the best of our knowledge, the component-variable biparticulate CuInSe_2_/ZnS MNC have not been prepared yet. In this work, we reported the first successful attempt on this objective concerning preparation of CuInSe_2_/ZnS nanocrystals with different intercomponent ratio, supplemented by second-step wet stirred media milling route using a circulation mill stabilizing these CuInSe_2_/ZnS nanocrystals in 0.5% SDS solution (the SDS-stabilized CuInSe_2_/ZnS nanosuspensions).

## 2. Materials and Methods

### 2.1. Mechanochemical Synthesis of CuInSe_2_/ZnS Nanocrystals and SDS Capped CuInSe_2_/ZnS Nanosuspensions

**Figure 1:**
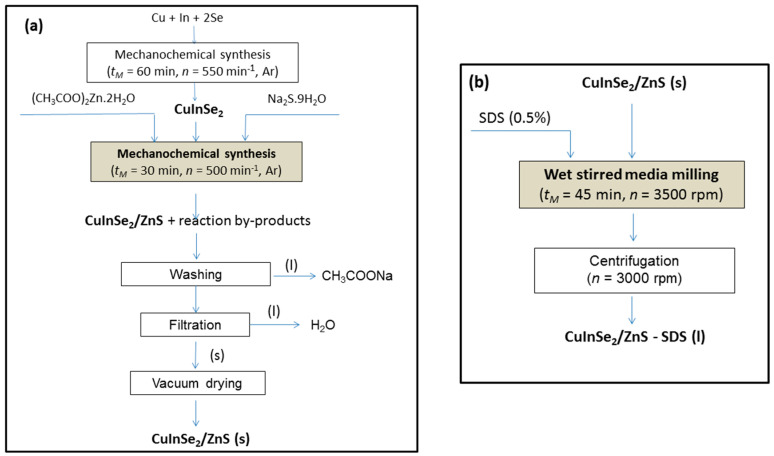
Flow-sheets of the technological routes to prepare CuInSe_2_/ZnS nanocrystals (**a**) and sodium dodecyl sulphate (SDS) capped CuInSe_2_/ZnS nanosuspensions (**b**).

The component-variable CuInSe_2_/ZnS MNC in a molar CuInSe_2_:ZnS ratio approaching 5:0, 4:1 and 1:4 (chosen at the basis of previous research for ZnS-based nanocrystals [15,17]) were prepared by co-milling of CuInSe_2_ (previously synthesized by milling from elemental ingredients purchased in Merck, Darmstadt, Germany, the 99.7% Cu, 99.99% In and 99.9999% Se, according the procedure described in [27]) and precursors for ZnS preparation (zinc acetate dihydrate, 99%, Ites, Vranov nad Topľou, Slovakia, and sodium sulfide nonahydrate, 98%, Acros Organics, NJ, USA), as it was described in more details elsewhere [28,29]. The respective preparation route for CuInSe_2_/ZnS nanocrystals is highlighted in Figure 1 (left), it obeyed the following reactions:

Cu + In + 2Se → CuInSe_2_ (1)

CuInSe_2_ + (CH_3_COO)_2_Zn∙2H_2_O + Na_2_S∙9H_2_O → CuInSe_2_/ZnS + 2CH_3_COONa + 11H_2_O (2)

Co-milling was performed in a planetary ball mill Pulverisette 6 (Fritsch, Idar-Oberstein, Germany) in an argon atmosphere for 30 min. The 250 mL tungsten carbide milling chamber with 50 tungsten carbide balls, having 10 mm in diameter was used. The rotational speed of the planet carrier n was 500 rpm. After the synthesis, the sodium acetate as side product of Equation (2), was removed by washing with distilled water. After vacuum drying (70 °C, 180 min), a solid phase of CuInSe_2_/ZnS nanocrystals was obtained.

In order to obtain colloidal form of nanocrystals suitable for testing of their biological activity, the wet stirred media milling route was applied. The previously prepared CuInSe_2_ and CuInSe_2_/ZnS nanocrystals were subjected to wet milling in the 0.5% SDS solution under the following conditions: 4 g of CuInSe_2_ or CuInSe_2_/ZnS in a total, 300 mL of SDS solution (0.5 wt.%) and 45 min milling at *n* = 3500 rpm. After milling, the samples were centrifuged at *n* = 3000 rpm. The scheme of preparation of CuInSe_2_ and CuInSe_2_/ZnS nanocrystals capped by SDS is illustrated on Figure 1 (right).

### 2.2. Characterization Methods

The phase-microstructure analysis of the prepared MNC were performed using X-ray powder diffraction (XRPD) method collected the data in a transmission mode on STOE STADI P diffractometer (STOE Automated DIffractometer for Powder, STOE & Cie GmbH, Darmstadt, Germany) with the following setup: CuK_α1_-radiation, curved Ge (111) monochromator on primary beam, linear position-sensitive detector and 2θ/ω-scan. Preliminary data processing was performed employing STOE WinXPOW [30] and Powder Cell [31,32] program packages, using crystallographic data taken from the known databases [33]. The crystal structures of the phases were refined by the Rietveld method with FullProf.2k program (version 5.60) [34,35]. Quantitative phase analysis according to [35] and microstructure parameters of the identified phases (average apparent crystallite size D in terms of size of coherently diffracting domains, and average maximum strain ε) were determined by isotropic line broadening analysis implemented in this program [36].

The room-temperature micro-Raman spectroscopic measurements were performed in backscattering geometry under the excitation from focused Ar laser beam (514 nm), using confocal Raman Microscope (Spectroscopy & Imaging, Warstein Germany). The Raman line of crystalline Si (520 cm^−1^) was employed to calibrate the system in the present study.

Transmission electron microscopy (TEM) was used to characterize the prepared MNC samples at a nanoscale. A small amount of sample was ultrasonically homogenized in absolute ethanol for 5 min. Then, a droplet of the suspension was applied onto a lacey carbon-coated nickel grid and dried. Prior to the TEM analyses, the samples were carbon-coated to prevent charging under the electron beam. The TEM analyses were performed using a 200 kV microscope JEM 2100 (JEOL, Akishima, Japan) with LaB_6_ electron source, this set-up being equipped with energy dispersive X-ray spectrometer (EDXS) for chemical analysis. The morphology of the MNC was investigated using field emission-scanning electron microscope (FE-SEM) Mira 3 (Tescan, Brno, Czech Republic) coupled with an EDXS analyzer (Oxford Instruments, Oxford, UK).

Adsorption isotherms and pore size distribution in the MNC samples were obtained using NOVA 1200e Surface Area & Pore Size Analyzer (Quantachrome Instruments, Hook, UK), the specific surface area and pore size distribution being calculated by respectively applying the Brunnauer–Emmet–Teller (BET) and Barret–Joyner–Halenda (BJH) methods.

Optical absorption spectra were recorded using UV-Vis spectrophotometer Helios Gamma (Thermo Electron Corporation, Cambridge, UK). The measurements were performed in quartz cell by dispersing the synthesized particles in absolute ethanol by ultrasonic stirring. The photoluminescence (PL) spectra were registered using UV-Vis-NIR confocal Raman Microscope (Spectroscopy & Imaging, Warstein, Germany) with 488 nm line of Ar laser for excitation. The samples were dispersed on SiO_2_/Si substrate for PL intensity measurement.

The particle size distribution was measured by a photon cross-correlation spectroscopy using a Nanophox particle size analyzer (Sympatec, Clausthal-Zellerfeld, Germany). A portion of nanosuspension was diluted with the BSA-containing solution to achieve a suitable concentration for the measurement. This analysis was performed using a dispersant refractive index of 1.33. The measurements were repeated three times for each sample.

Zeta-potential (ZP) was registered for the samples diluted in a distilled water using Zetasizer Nano ZS (Malvern, Malvern, UK) set-up, the electrophoretic mobility of the particles being converted to ZP using the Smoluchowski equation built in the Malvern zetasizer software. These ZP measurements were performed in triplicate with at least 12 sub-runs for each sample.

Fourier transform infrared (FT-IR) in transmission mode was performed using a Tensor 27 spectrometer (Bruker, Karlsruhe, Germany). The samples were prepared by a KBr-pellet method and measured in the frequency range of 4000–400 cm^−1^. KBr was dried before the analysis at 100 °C for 1 h. The spectra were expressed as absorbance versus wavenumber (cm^−1^).

Dissolution tests were conducted in 250 mL glass reactor under the following conditions: The weight of the sample—0.5 g, the volume of the physiological solution (0.9% NaCl)—200 mL and temperature—37 ± 0.5 °C. Aliquots (1 mL) and diluted as necessary of the solution were collected at appropriate intervals for the determination of the dissolved copper and zinc by the atomic absorption spectroscopy (AAS).

The content of metal ions in solid samples was analyzed using an atomic absorption spectrometer SPECTRAA L40/FS (Varian, Crawley, Australia).

### 2.3. Biological Activity

#### 2.3.1. Multiple Myeloma Cell Lines

Multiple myeloma (MM) cell lines (MM.1S, RPMI 8226-S (referred to as RPMI-S), OPM-1, OPM-2, KMS-11 and JJN3 were obtained from ATCC (Manassas, VA, USA). MM cell lines were cultured in RPMI 1640 (Cellgro, Mediatech, VA, USA) supplemented with 10% heat-inactivated fetal bovine serum (FBS; Harlan, Indianapolis, IN, USA), 100 U/mL penicillin, 100 μg/mL streptomycin and 2 mM L-glutamine (GIBCO, Grand Island, NY, USA) at 37 °C in 5% CO_2_, respectively.

#### 2.3.2. Nanoparticles Sensitivity by Cell-Based MTT Assay

The MM cell lines were plated in 96-well plates at a density of 1 × 10^4^ cells per well, and treated with increasing concentrations (0–10 µM) of CuInSe_2_/ZnS at ratio 5:0 and 4:1 for 24 h, 48 h and 72 h compared to control cells treated with same concentration of SDS as in nanoparticles.

The cytotoxic effect was determined by 50 µL per well of the 3-(4,5-dimethylthiazol-2-yl)-2,5-diphenyltetrazolium bromide assay (MTT; 1 mg/mL, Sigma-Aldrich, St Louis, MO, USA) for 4 h. Formazan crystals were dissolved with addition of 150 µL of DMSO, and absorbance was measured at 540 and 690 nm in a spectrophotometer (xMark™ Microplate Absorbance Spectrophotometer, Biorad, California, CA, USA). The concentration of nanoparticles that inhibited cell survival to 50% (EC50) was determined by Calcusyn software (Biosoft, Ferguson, MO, USA).

#### 2.3.3. Nanoparticle Sensitivity by Flow Cytometry Analysis

The MM cell lines were plated in 12-well plates at a density of 2 × 10^5^ cells per well, and treated with CuInSe_2_/ZnS at ratio 5:0 and 4:1 at 5 µM concentration for 24 h. Briefly, both suspension and adherent cells were collected and washed with cold PBS. Cells were resuspended in 400 µL of PBS and 7-AAD (Molecular probes, Eugene, OR, USA; final concentration = 1 μg/mL) to gate out dead cells. After 15 min incubation in the dark at room temperature, cells were analyzed by a FACS Aria Special Sorter equipped with UV laser (Becton Dickinson, Mountain View, CA, USA). The nanoparticles were excited at violet (405 nm) laser and emitted by 670+/−30 nm wavelength.

## 3. Results and Discussion

### 3.1. Characterization of CuInSe_2_/ZnS Nanocrystals

**Figure 2:**
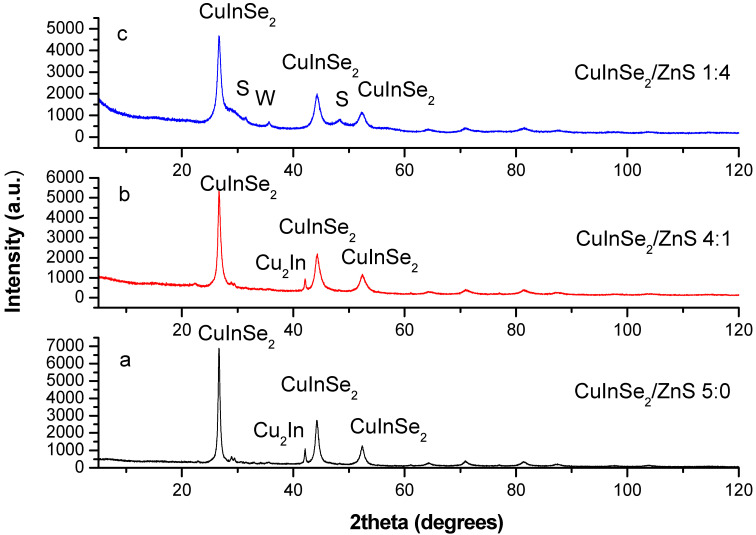
Observed X-ray powder diffraction (XRPD) patterns of CuInSe_2_/ZnS MNC: (**a**) CuInSe_2_/ZnS 5:0, (**b**) CuInSe_2_/ZnS 4:1 and (**c**) CuInSe_2_/ZnS 1:4 (the ZnS phase is denoted as S—sphalerite, and W—wurtzite).

**Figure 3:**
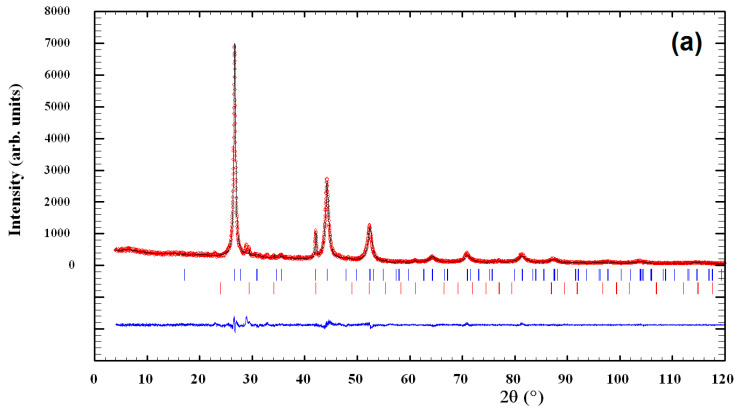

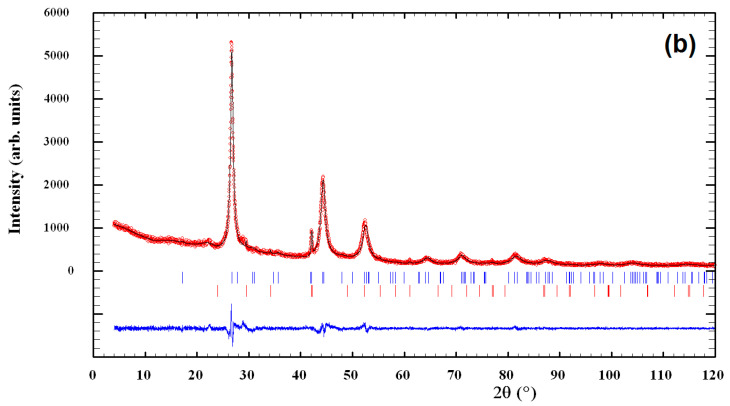
Observed and calculated XRPD profiles for CuInSe_2_/ZnS 5:0 (**a**) and CuInSe_2_/ZnS 4:1 (**b**) multinanocomposites (MNC). Experimental data (circles) and calculated profile (solid line) are given with calculated Bragg positions for CuInSe_2_ (the upper raw of blue vertical ticks), Cu_2_In (the bottom raw of red vertical ticks) and difference curve (the bottom solid line). The presence of the third phase with unknown crystal structure in both samples is possible (see text for more details).

**Figure 4:**
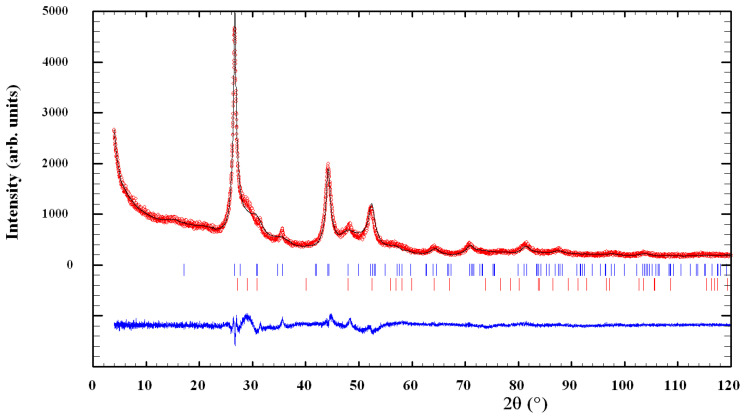
Observed and calculated XRPD profiles for CuInSe_2_/ZnS 1:4 MNC. Experimental data (circles) and calculated profile (solid line) are given with calculated Bragg positions for CuInSe_2_ (the upper raw of blue vertical ticks), ZnS wurtzite (the bottom raw of red vertical ticks) and difference curve (the bottom solid line). The presence of ZnS sphalerite phase is also possible (see text for more details).

The XRPD patterns of mechanosynthesized CuInSe_2_/ZnS nanocrystals with different intercomponent ratio 5:0, 4:1 and 1:4 are compared in Figure 2, Figure 3 and Figure 4.

The CuInSe_2_/ZnS 5:0 sample consists of three different phases (see Figure 2a and Figure 3a). The main phase (94.7(6) mass.%) is CuInSe_2_: Structure type CuFeS_2_, space group I-42d, unit cell parameters *a* = 5.782(3) and *c* = 11.583(11) Å. Additional phase (5.3(1) mass.%) is Cu_2_In: Structure type Co_1.75_Ge, space group P6_3_/mmc, unit cell parameters *a* = 4.2888(6) and *c* = 5.2457(14) Å. Small admixture of the third phase with maximal reflex at 2*θ* ~ 22.93° is also detectable, the distribution of the respective reflexes being like to Cu_2_TeSe_4_ phase (JCPDS card No. 27-0186). With acceptance of analogy between Se and Te, it seems reasonable that this phase is Cu_2_Se_5_ with smaller unit cell parameters. Since the crystal structure of this phase is unknown, it was not accepted during refinement. Thus, the content of main phase CuInSe_2_ was determined as close to ~90 mass.%. The estimated numerical value of average apparent crystallite size D for CuInSe_2_ approaches 9.8 ± 2.9 nm, while average maximum microstrain ε achieves 1.20(3)%.

The CuInSe_2_/ZnS 4:1 sample is also three-phased one (see Figure 2b and Figure 3b), the phase composition being identical to the previous. The unit cell parameters for the main (96.8(6) mass %) CuInSe_2_ phase are *a* = 5.7582(12) and *c* = 11.616(4) Å, and microstructural parameters are *D* = 7.1 ± 1.8 nm and ε = 1.67(8)%. The ZnS phase in this sample is probably in highly dispersive state, since there are no visible relaxations from wurtzite and/or sphalerite phases.

In case of CuInSe_2_/ZnS 1:4 sample (Figure 2c and Figure 4), the main phase (53.8(3) mass. %) is CuInSe_2_ with unit cell parameters *a* = 5.7696(14) and *c* = 11.642(5) Å, and microstructural parameters *D* = 6.4 ± 4.3 nm and ε = 1.80(4)%. Additional phase in this sample is high-temperature ZnS modification preferentially with hexagonal wurtzite structure. The presence of room-temperature ZnS modification with cubic sphalerite structure is also possible, but more reliable identification is difficult in view of semi-amorphous type of the collected XRPD pattern (the overlapped reflections).

**Figure 5:**
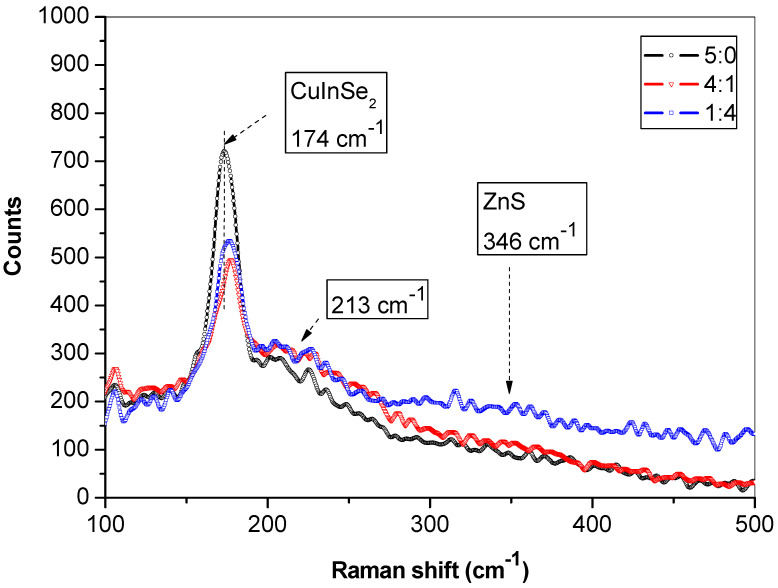
Raman spectra of CuInSe_2_/ZnS MNC excited at 514 nm Ar laser beam.

The Raman spectra from mechanochemically synthesized CuInSe_2_/ZnS nanocrystals excited by Ar laser beam (514 nm) are shown in Figure 5. The main part of the spectrum is located between 100 and 300 cm^−1^. For all measured samples, the ternary CuInSe_2_ phase is clearly identified by two peaks at 174 and 213 cm^−1^. The most intense peak at 174 cm^−1^ is due to the characteristic A_1_ mode of the chalcopyrite CuInSe_2_ phase [37,38,39]. The blue shift in the position of this A_1_ mode (as compared with announced in [37,38]) is probably due to nanocrystalline structure of this sample, and it could be related to the presence of high density of structural defects in the scattering volume. This specificity constitutes the main vibrational mode from chalcopyrite-ordered CuInSe_2_, allowing additional modes in the spectral region between 173 and 216 cm^−1^ (B1, B2, 2E) [37]. The 174 cm^−1^ peak intensity in CuInSe_2_ sample [39] is stronger than in CuInSe_2_/ZnS 4:1 and CuInSe_2_/ZnS 1:4 MNC samples. The peaks ascribed to A_1_ mode in CuInSe_2_/ZnS nanocrystals are red shifted in comparison with these peaks in CuInSe_2_ alone. The very weak peak ascribed to E vibrational mode in CuInSe_2_ is located at 213 cm^−1^.

It can be seen some minor changes in the Raman spectra for samples before and after mixing with ZnS. For sample with molar ratio 1:4, the broader peak at 335–340 cm^−1^ can be assigned to LO modes of A_1_ and E_1_ symmetry (351 cm^−1^ [40]), and mixed prevailing surface optical (SO) mode of ZnS (335 cm^−1^). This is in accordance with the results discussed in previous studies on ZnS nanowires [41,42], where SO phonon mode varies in wavenumber depending on the shape and surface roughness of ZnS nanostructures.

**Figure 6:**
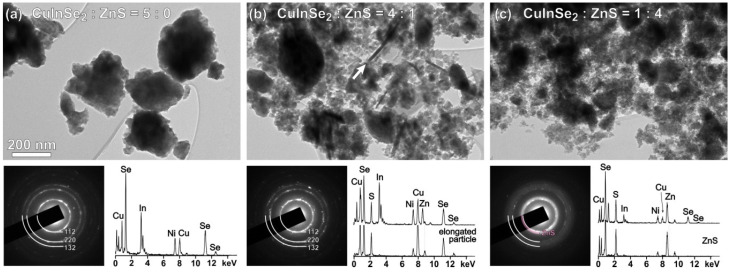
Low-magnification images of CuInSe_2_/ZnS MNC: (**a**) CuInSe_2_/ZnS 5:0, (**b**) CuInSe_2_/ZnS 4:1 and (**c**) CuInSe_2_/ZnS 1:4 with selected area electron diffraction (SAED) patterns and energy dispersive X-ray spectrometer (EDXS) spectra recorded at characteristic parts of the samples.

The prepared CuInSe_2_/ZnS MNC samples were further characterized by TEM method, the low-magnification images of three samples along with selected area electron diffraction (SAED) patterns and results of EDXS analyses being presented in Figure 6.

The CuInSe_2_ sample (Figure 6a) is composed of agglomerated, randomly oriented CuInSe_2_ nanoparticles exhibiting good crystallinity, as it follows from sharp diffraction rings of the respective SAED pattern. Several EDXS analyses performed in different parts of the sample revealed relatively homogenous composition of the sample with elemental ratio close to the expected Cu:In:Se = 1:1:2. Due to small amount, two secondary phases determined from XRPD analyses of this sample (Figure 2a and Figure 3a) were not detected by TEM.

In CuInSe_2_/ZnS 4:1 MNC sample (Figure 6b), the ZnS nanoparticles are present in the form of extremely fine crystallites surrounding larger CuInSe_2_ agglomerates as previously observed for CuInS_2_/ZnS system [43]. The presence of ZnS nanoparticles is barely observed in the SAED pattern, whereas the presence of Zn and S from ZnS nanoparticles surrounding the initial CuInSe_2_ agglomerates is clearly evident from EDXS. In this sample, the small amount of nanoparticles with elongated morphology was observed. In respect to EDXS analysis, these nanoparticles are shown to be composed of Cu, S and Se. It seems these nanoparticles are formed by reaction between one of the secondary phases in CuInSe_2_ detected by XRPD, e.g., Cu_2_Se_5_ and sodium sulfide nonahydrate added to form ZnS.

In MNC sample with highest ZnS fraction (CuInSe_2_/ZnS 1:4), the presence of ZnS nanocrystallites is clearly observed in the SAED pattern shown in Figure 6c. Due to extremely small crystallite size (<5nm), the ZnS phase yields diffuse diffraction rings, making impossible distinction between sphalerite and wurtzite polymorphs. According to HRTEM analysis of CuInS_2_/ZnS [43], the ZnS component is mainly stabilized as sphalerite phase, with many defects having locally wurtzite-type stacking (stacking faults and twin boundaries).

The specific surface area *S_BET_* of pure CuInSe_2_ MNC sample is 3.4 m^2^/g, the value which is more-or-less anticipated as for mechanochemically activated chalcogenides [15,16,17]. Upon further ZnS introduction (in CuInSe_2_/ZnS 4:1), this parameter is increased to 21 m^2^/g. The sample with highest ZnS content exhibits the *S_BET_* value approaching as high as 108 m^2^/g. In our previous study, the *S_BET_* values for the CuInS_2_/ZnS system prepared in a similar manner were reported to be 6 and 86 m^2^/g for ZnS-free and ZnS-rich compounds, respectively [43]. Whereas the as-synthesized CuInSe_2_ exhibits almost two-fold lower *S_BET_* value than CuInS_2_, the CuInSe_2_/ZnS 1:4 MNC sample has higher *S_BET_* than the corresponding sulfide analogue. Maybe, the less porous structure of selenide MNC with respect to the corresponding sulfide offers the possibility of ZnS to manifest its porosity better by being exposed to the nitrogen gas on flat surface. In the case of sulfide, the ZnS particles can be trapped inside already present pores of CuInS_2_ and the gas cannot reach them so easily. In the report applying solvothermal approach for CuInSe_2_ nanoparticles, significantly higher values (ca. 8.22 and 44.8 m^2^/g for quantum dots and dandelion-like particles, respectively) were reported [44]. This can be caused by applied method. In solvothermal method, the porous crystals have enough time to grow, whereas in mechanochemical synthesis, the porous structure is immediately brought down by heavy impacts of milling balls.

**Figure 7:**
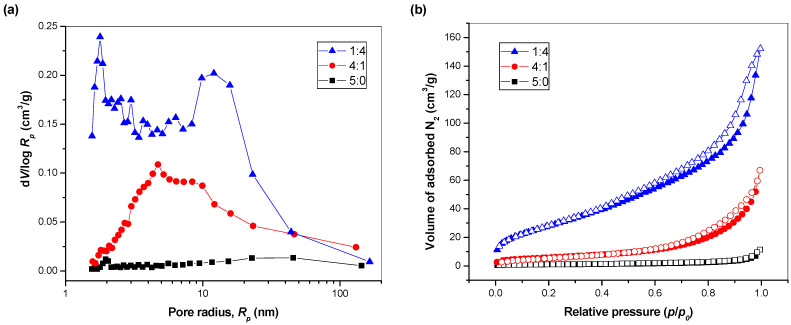
Surface properties of the synthesized MNC: (**a**) adsorption–desorption isotherms and (**b**) pore size distributions.

To study the surface properties of mechanochemically synthesized CuInSe_2_/ZnS nanocrystals in more details, the whole adsorption–desorption isotherms and particle size distributions for these samples were analyzed (see Figure 7).

The adsorption isotherm of CuInSe_2_ sample resembles that of non-porous solid, although the area around relative pressures of one hints to the presence of macropores. This is confirmed by pore size distribution of this sample shown in Figure 7b. Almost similar situation was evidenced for CuInS_2_ earlier [43].

Upon ZnS introduction, the mesopores are formed, as evidenced by hysteresis loops in isotherms in both ZnS-containing samples in Figure 7a. The CuInSe_2_:ZnS 4:1 nanocrystals have broad pore size distribution covering almost the whole mesoporous range with the maximum around 4 nm. Macropores are also present, which are most probably coming both from CuInSe_2_ and ZnS. When the content of ZnS is increased further (as in CuInSe_2_:ZnS 1:4 sample), the porous properties are improved more (and also very small mesopores with maximum radius around 2 nm are formed). This is not an artifact, as such a peak, also more diffuse, was evidenced upon calculation from adsorption isotherm (not shown here). In addition to this maximum, another one around 15 nm radius is present. The pore size distribution of this sample resembles that reported for pure ZnS in [43] rather than for CuInS_2_:ZnS 1:4, in which the fraction of larger mesopores was missing. This is supported also by higher *S_BET_* value of CuInSe_2_:ZnS 1:4 than CuInS_2_:ZnS 1:4 sample discussed earlier. The changes observed with the introduction of ZnS suggest that the prepared sample is not just a pure mixture of both sulfides, but some interactions at least on the level of the Van der Waals forces are definitely important.

**Figure 8:**
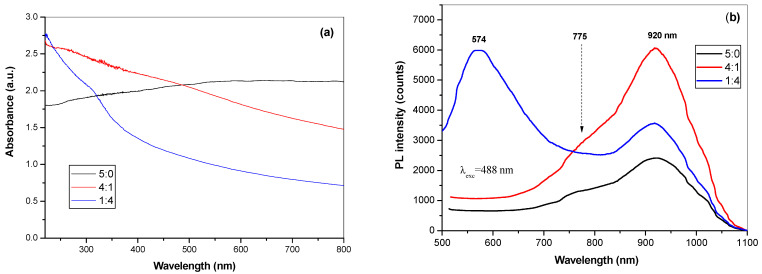
(**a**) UV-Vis and (**b**) micro-photoluminescence (PL) (**b**) spectra of the synthesized CuInSe_2_/ZnS 5:0, CuInSe_2_/ZnS 4:1 and CuInSe_2_/ZnS 1:4 MNC excited at 488 nm.

Optical properties of mechanochemically synthesized CuInSe_2_/ZnS nanocrystals were recorded by UV-Vis and micro-PL spectroscopy, the spectra being shown in Figure 8.

By incorporation of ZnS into CuInSe_2_, the absorption spectra of CuInSe_2_/ZnS 4:1 and CuInSe_2_/ZnS 1:4 MNC show absorption peak at 320 nm (3.85 eV) typical for ZnS, which is blue shifted due to quantum confinement effect in respect to the bulk band gap of ZnS [5].

The room temperature PL spectra excited with the wavelength of 488 nm are presented in Figure 8b. There are two band emissions for CuInSe_2_ phase, these being weak emission in visible range at 775 nm (1.6 eV) and more intensive emission at 920 nm (1.34 eV). The emission spectrum is red-shifted by 0.29 eV respectively to the band edge. The strong emission at 1.34 eV appears is due to excitonic band-to-band (e-h) recombination, while peak at 1.6 eV is attributed to transition from shallow trap centers to conduction band. For CuInSe_2_/ZnS 1:4 MNC sample, the broad green emission spectra are observed due to some self-activated defect centers related to Zn and S-vacancies [45].

### 3.2. Characterization of SDS Capped CuInSe_2_/ZnS Nanosuspensions

To obtain colloidal CuInSe_2_/ZnS nanocrystals dispersed in SDS solution suitable for their biological and anti-cancer testing, the wet stirred media milling process was applied, the respective MNC samples being further referred to as CuInSe_2_/ZnS-SDS. We tried to prepare nanosuspensions for all three above studied CuInSe_2_/ZnS nanocrystals (5:0, 4:1 and 1:4). Unfortunately, we have only prepared SDS capped CuInSe_2_/ZnS 5:0 and CuInSe_2_/ZnS 4:1 nanosuspensions. Therefore, only these nanosuspensions were further characterized.

**Figure 9:**
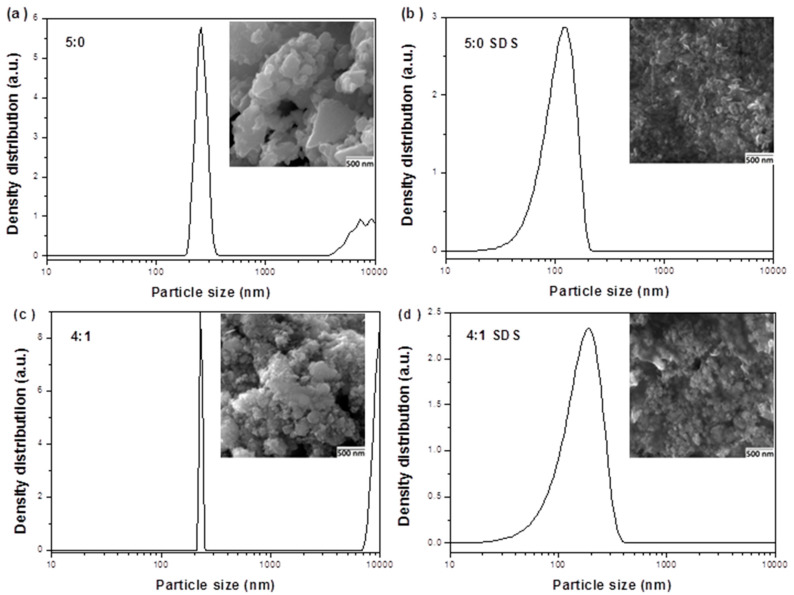
Particle size distribution in CuInSe_2_/ZnS nanocrystals (**a**,**c**) and CuInSe_2_/ZnS-SDS nanosuspensions (**b**,**d**) with intercomponent ratio 5:0 (**a**,**b**) and 4:1 (**c**,**d**). The inset shows representative SEM image of the MNC sample.

Changes in particle size distribution during such processing are displayed in Figure 9 with morphology of the samples documented by SEM micrographs in the insets. As can be seen, the particle size distributions were changed from polymodal (Figure 9a,c) to unimodal after wet milling in SDS (Figure 9b,d). The greatest particles (>1 µm) disappear after wet milling, being transformed into the finest ones (see Figure 9b,d). The SDS on the surface of these particles was sufficient to separate them and avoid aggregation. In the case of CuInSe_2_/ZnS 5:0 nanocrystals, the particle size median d_50_ was 269 nm, and after wet milling the obtained suspension was getting gradually smaller with d_50_ = 123 nm. The similar situation was characteristic for CuInSe_2_/ZnS 4:1 nanocrystals with *d*_50_ gradually dropped under wet milling (from *d*_50_ = 227 nm to *d*_50_ = 188 nm).

The above results are in accordance with representative SEM images of these samples shown as insets in Figure 9. The SEM images of CuInSe_2_/ZnS nanocrystals before wet milling (Figure 9a,c) manifest, that powder samples are composed of fine nanoparticles creating densely packed irregular aggregates. Besides larger micrograins, great amount of nanosized grains (200–300 nm in sizes) can be found. Noteworthy, the studied CuInSe_2_/ZnS-SDS samples contain also a large portion of homogeneously distributed nanocrystallites. It follows from SEM images that in the case of samples capped with SDS (CuInSe_2_/ZnS-SDS 5:0 and 4:1, Figure 9b,d) the particles are smaller than ones in samples without SDS (CuInSe_2_/ZnS 5:0 and 4:1, Figure 9a,c).

The results of ZP measurements for these MNC before and after milling in SDS are summarized in Table 1. As can be seen, both 5:0 and 4:1 samples were dispersed in distilled water have negative ZP, belonging to unstable area close to zero (−19 and −6.2 mV, respectively). Therefore, the anionic surfactant SDS was used to improve their stability. After 45 min milling of the nanocrystals in this surfactant, the ZP values were shifted to more negative ones (−41 and −39 mV, respectively), to the areas of the better stability. The obtained unimodal particle size distributions correlate very well with these ZP values.

**Figure 10:**
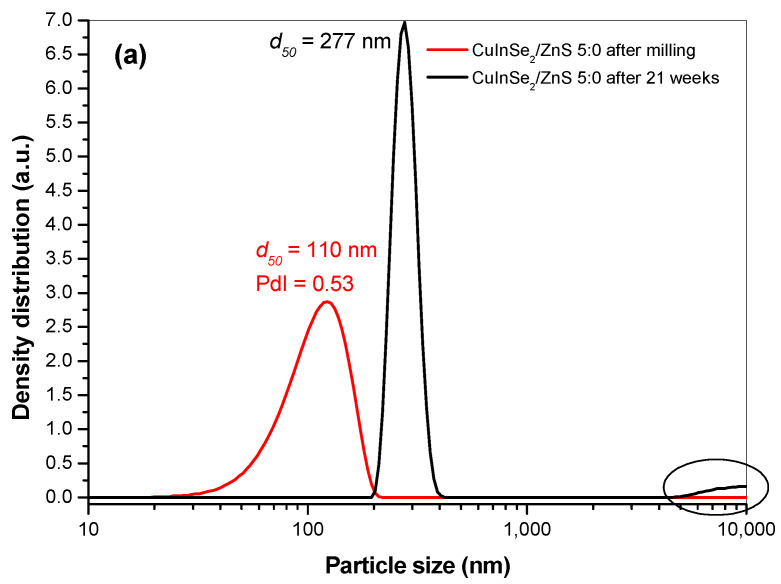

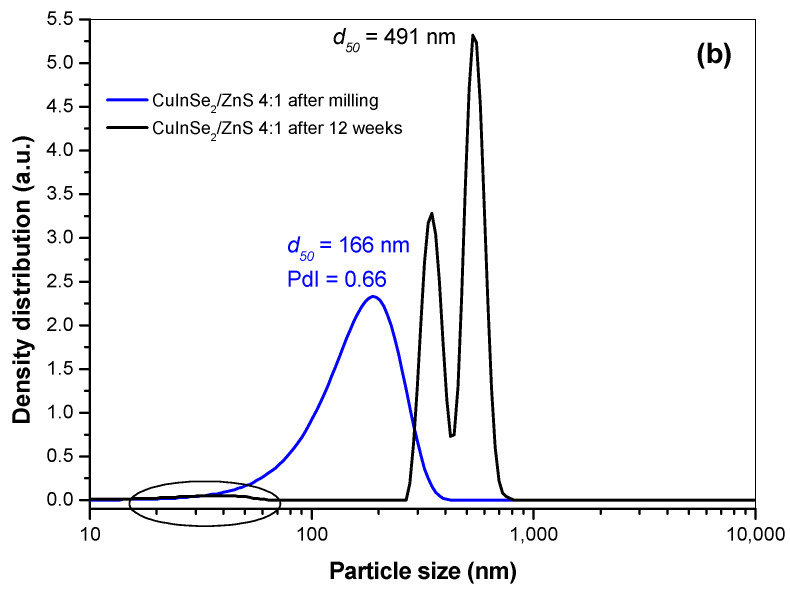
Changes in particle size distribution in the nanosuspensions CuInSe_2_/ZnS 5:0-SDS (**a**) and CuInSe_2_/ZnS 4:1-SDS (**b**) during prolonged storage.

The long-term stability of the prepared CuInSe_2_/ZnS-SDS nanosuspensions was also studied using respective particle size distributions measured after prolonged storage (Figure 10). It was found that CuInSe_2_/ZnS 5:0-SDS nanosuspension was stable for 20 weeks, whereas CuInSe_2_/ZnS 4:1-SDS was stable for about half time shorter (11 weeks).

This difference can be reasonably explained by deep analysis of the respective particle size distributions measured after MNC milling in surfactant (red and blue curves on Figure 9b,d and Figure 10a,b) and comparison with the calculated polydispersity index (PdI). In case of CuInSe_2_/ZnS 5:0-SDS nanosuspension (Figure 10a, red curve), the particle size distribution is narrower (from 20 to 200 nm) with PdI value approaching 0.53. On the other hand, the particle size distribution in CuInSe_2_/ZnS 4:1-SDS nanosuspension (Figure 10b, blue curve) is wider (from 10 to 400 nm) with PdI approaching 0.66. Thereby, the higher PdI, the higher polydispersity, and, consequently, the lower stability of the system. In final, this effect leads to Ostwald ripening process, where the coarse-grained particles grow at the expense of the fine-grained particles [46], which are more soluble thus allowing mass transfer towards less soluble coarse-grained particles [24]. These structural transformations result in particles aggregation (see Figure 10a,b, black curves).

**Figure 11:**
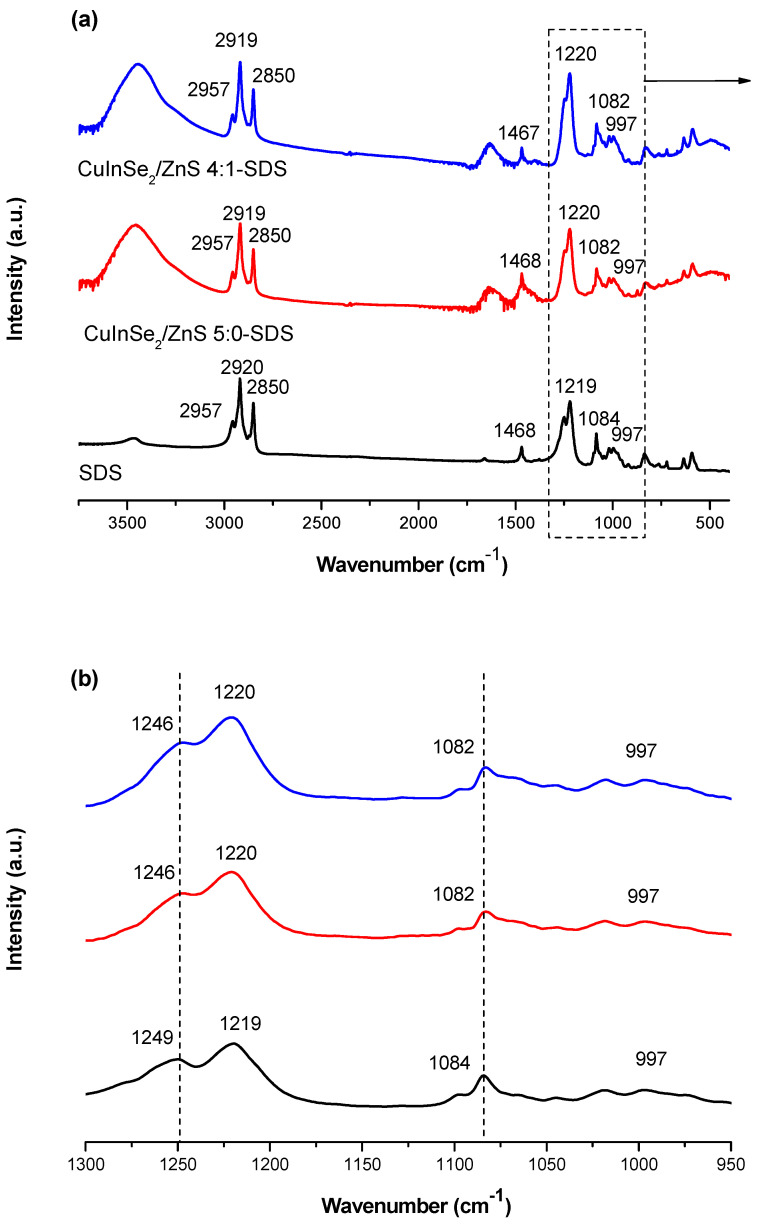
FTIR spectra of SDS (**black**), SDS capped nanosuspensions CuInSe_2_/ZnS 5:0-SDS (**red**) and CuInSe_2_/ZnS 4:1-SDS (**blue**). (**a**) the whole spectrum; (**b**) a characteristic region for SO_2_ vibrations of SDS.

To confirm interaction between CuInSe_2_/ZnS and SDS, the ATR-FTIR spectra (in the range of 4000–400 cm^−1^) of SDS and SDS capped CuInSe_2_/ZnS-SDS nanosuspensions were recorded (see Figure 11). The spectrum of pure SDS (Figure 11a, black) contains two major regions attributing to aliphatic group of hydrophobic tail (3000–2800 cm^−1^) and sulfonic acid group of hydrophilic head (1250–950 cm^−1^) [47]. The spectrum also exhibits characteristic bands ascribed to O–H stretching (3472 cm^−1^) and CH_2_ scissoring vibrations in hydrocarbon segment (1468 cm^−1^). The region 3000–2800 cm^−1^ is attributed to C-H stretching containing asymmetric (2957 cm^−1^) and symmetric (2850 cm^−1^) CH_3_ and asymmetric CH_2_ (2920 cm^−1^) vibrational modes. The region 1250–950 cm^−1^ is attributed to asymmetric (1220 and 1249 cm^−1^) and symmetric (1084 cm^−1^) SO_2_ vibrational modes [48].

After capping of CuInSe_2_/ZnS nanoparticles with SDS negligible shifts were detected in spectra. In the region of C–H symmetric and asymmetric stretching vibrations modes of aliphatic group of hydrophobic tail (3000–2800 cm^−1^ and 1468 cm^−1^) definitely no changes were registered. However, in S=O stretching region of the sulfonic acid group of hydrophilic head slight shifts (from 1249 to 1246 cm^−1^ and from 1219 to 1220 cm^−1^) and changes in shape of spectra were detected. Therefore, it shows that the capping could be due to negatively charged head group moieties (Figure 11b, red). Moreover, a number of references mentioning the presence of adsorption bands of SDS at similar places have been introduced [49,50,51].

### 3.3. Biological Activity of SDS Capped CuInSe_2_/ZnS 5:0 and 4:1 Nanosuspensions

#### 3.3.1. Dissolution of Copper and Zinc from CuInSe_2_/ZnS Nanocrystals

**Figure 12:**
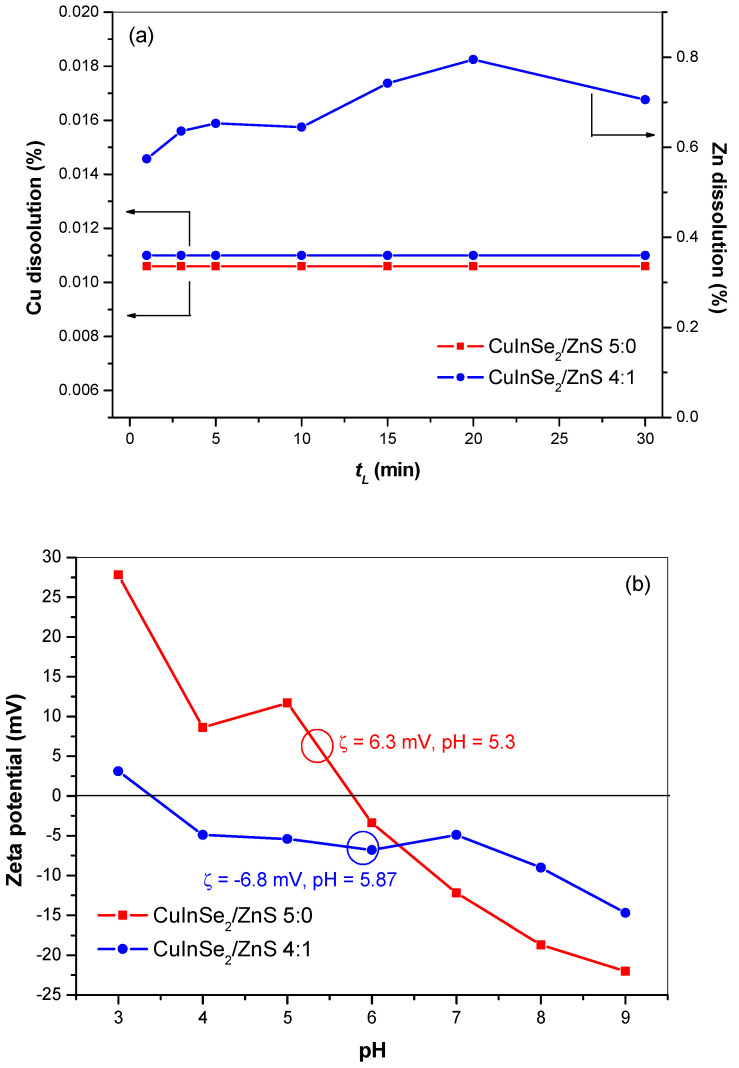
The dissolution of Cu(II) and Zn(II) ions (**a**) and ZP measurements (**b**) of CuInSe_2_/ZnS 5:0 and CuInSe_2_/ZnS 4:1 nanocrystals.

As one of possible applications of CuInSe_2_/ZnS MNC is their usage in nanomedicine. The results of metal (Cu and Zn) dissolution testing in CuInSe_2_/ZnS 5:0 and CuInSe_2_/ZnS 4:1 nanocrystals are presented in Figure 12. The dissolution was performed in a physiological medium (0.9% NaCl solution) at human body temperature (37 ± 0.5 °C) for 30 min. As seen, the Cu dissolution for both MNC samples is very low (~0.011%). In the case of CuInSe_2_/ZnS 4:1 sample, the dissolution of Zn was reached as high as 0.7% (Figure 12a). These results can be confronted with data on ZP measurements shown in Figure 12b.

For the better understanding of the differences in metals dissolution which were occurred during dissolution, we tried to explain this dissolution phenomena by means of ZP. After addition of CuInSe_2_/ZnS 5:0 sample into physiological medium, the pH value was 5.3 and positive ZP = 6.3 mV was detected. According to the XRPD data (Figure 3a), this sample prepared by mechanochemical route possesses chalcopyrite crystal structure, in which each S(-II) anion is tetrahedrally coordinated to two Cu(I) cations and two In(III) cations. The positive ZP value is consequence of Cu(I) and In(III) cations contribution at crystal surface. The slight Cu dissolution was obtained from the surface of a sample. However, addition of CuInSe_2_/ZnS 4:1 sample into the physiological medium, brings about the pH value as 5.87. Contrary to CuInSe_2_/ZnS 5:0 sample, the negative ZP (−6.8 mV) is obtained in this sample. Based on the SEM analysis and leaching experiments (higher Zn dissolution), we suppose the ZnS particles are on the surface or between CuInSe_2_ crystallites. The negative ZP is a consequence of sulfur ions excess (due to the presence of ZnS) on the surface of nanocrystals prepared by co-milling of CuInSe_2_ with ZnS precursors. Three possible scenarios could be realized during this process, these being surface reconstruction, interdiffusion of Zn atoms or cation exchange in the surface of CuInSe_2_. Therefore, the negative charge is reached, like in our previous study on CuInS_2_/ZnS mechanosynthesis [43].

#### 3.3.2. Cytotoxicity of CuInSe_2_/ZnS 5:0-SDS and CuInSe_2_/ZnS 4:1-SDS Nanosuspensions

**Figure 13:**
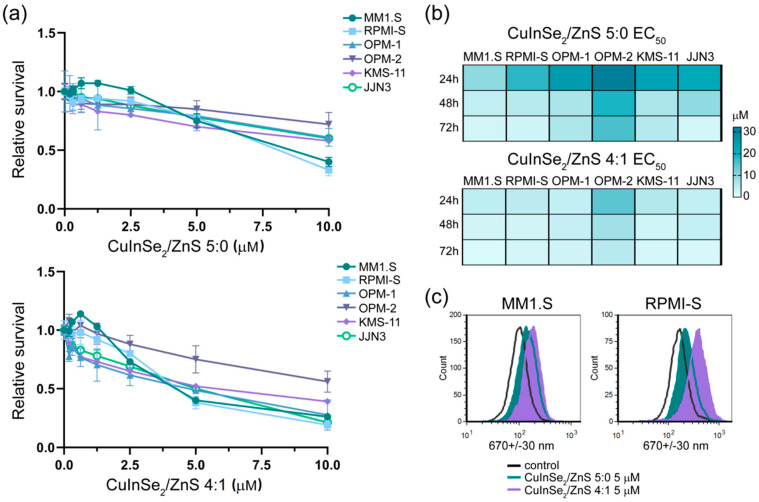
The multiple myeloma (MM) cell lines sensitized by CuInSe_2_/ZnS 5:0-SDS and CuInSe_2_/ZnS 4:1-SDS nanosuspensions. (**a**) MM cell lines (MM.1S, RPMI-S, OPM-1, OPM-2, KMS-11, and JJN3) were treated (0–10 μM) with CuInSe_2_/ZnS 5:0 (top) and CuInSe_2_/ZnS 4:1 (bottom) for 24 h. The viability was assessed by 3-(4,5-dimethylthiazol-2-yl)-2,5-diphenyltetrazolium bromide assay (MTT) assay. Each treatment with a specific concentration of the nanoparticle was done in triplicate. The data presented are mean ± standard error, and expressed as viability relative to untreated controls. (**b**) the *EC*_50_ value of CuInSe_2_/ZnS 5:0 and CuInSe_2_/ZnS 4:1 was determined for each MM cell line for 24 h, 48 h and 72 h by the CalcuSyn software and depicted by heatmap. (**c**) fluorescence of nanoparticles CuInSe_2_/ZnS (5 μM concentration) 5:0 (blue) and 4:1 (purple) compared to control untreated cell (unfilled) in MM1.S and RPMI-S cell lines showed by histogram. Fluorescent intensity was excited by violet (405 nm) laser and emitted by 670+/−30 nm wavelength by a FACS Aria Special Sorter.

To evaluate cytotoxic effects of composite nanoparticles, six MM cell lines (in part, MM.1S, RPMI-S, OPM-1, OPM-2, KMS-11 and JJN3) were treated by CuInSe_2_/ZnS 5:0-SDS and CuInSe_2_/ZnS 4:1-SDS nanosuspensions (1–10 μmol/L) and cell survival was determined by MTT assay. For 24 h, the stronger anti-MM cytotoxic effect was achieved with CuInSe_2_/ZnS 4:1-SDS nanosuspension (see Figure 13a). The concentration- and time-dependent reduced cell viability was observed by EC_50_ value (the concentration reducing cell survival by 50%) for both nanosuspensions for 24 h, 48 h and 72 h by the CalcuSyn software (Figure 13b). Similarly, the higher cytotoxic effects on MM cell lines were determined by CuInSe_2_/ZnS 4:1-SDS nanosuspension. The effect was two to six times and one to three times stronger at 24 h and 48/72 h, respectively, as compared to CuInSe_2_/ZnS 5:0-SDS nanosuspension. Comparing MM cell lines, the anti-MM sensitizing potential was similar on all tested MM cell lines with exception of OPM-2 cells which were more resistant to both samples and OPM-1 cells which were also resistant to CuInSe_2_/ZnS 5:0-SDS nanosuspension. This finding is in accordance with previous data determining more resistant effect of As_4_S_4_ nanoparticles on OPM-2 cells in comparison with other MM cells [52]. In summary, both CuInSe_2_/ZnS-SDS nanosuspensions show cytotoxic potential, with stronger anti-MM effect caused by CuInSe_2_/ZnS 4:1-SDS nanosuspension.

To confirm fluorescent activity of the MNC, we evaluate the fluorescent intensity of both CuInSe_2_/ZnS-SDS samples in viable MM cells (MM1.S and RPMI-S) by flow cytometry (Figure 13c). These samples show significant shift in fluorescence intensity determined by violet laser excitation and emission of 670+/−30 nm wavelength, whereas stronger fluorescence is determined by CuInSe_2_/ZnS 4:1 MNC. This observation not only proves MNC localization inside viable cells, but also supports idea of their usage as imaging agents or labeled quantum dots in biomedical applications as drug carriers at lower (not cytotoxic) concentrations.

## 4. Conclusions

Within this work, the synthesis of CuInSe_2_/ZnS multiparticulate nanocomposites by milling in a planetary ball mill via simple solid-state approach is first reported. The sodium dodecyl sulphate capped CuInSe_2_/ZnS nanosuspensions were prepared by wet stirred media milling to obtain stable suspensions suitable for bioimaging applications. However, it was not possible to prepare stable SDS capped CuInSe_2_/ZnS 1:4 nanosuspension. Therefore, only SDS capped CuInSe_2_/ZnS 5:0 and 4:1 nanosuspenions were further characterized. The CuInSe_2_/ZnS 5:0-SDS nanosuspension was shown to be stable for 20 weeks, whereas the CuInSe_2_/ZnS 4:1-SDS one was stable for about 11 weeks. After SDS capping, unimodal particle size distribution was obtained with particle sizes medians approaching, respectively, 123 nm and 188 nm for CuInSe_2_/ZnS 5:0-SDS and CuInSe_2_/ZnS 4:1-SDS nanocomposites. Successful stabilization of the prepared nanosuspensions due to SDS covering the surface of the nanocomposite particles was confirmed by zeta potential measurements. The prepared CuInSe_2_/ZnS 5:0-SDS and CuInSe_2_/ZnS 4:1-SDS nanosuspensions possessed anti-myeloma sensitizing potential assessed by significantly reduced viability of multiple myeloma cell lines, with efficient fluorescence inside viable cells and higher cytotoxic efficacy in CuInSe_2_/ZnS 4:1-SDS nanosuspension.

## Figures and Tables

**Table 1:** Zeta potential of CuInSe_2_/ZnS 5:0 and CuInSe_2_/ZnS 4:1 MNC samples measured in water (before milling) and in SDS (after wet milling).

Sample	*ξ* (mV)	pH
CuInSe_2_/ZnS 5:0-H_2_O	−19	7.00
CuInSe_2_/ZnS 5:0-SDS	−41	7.96
CuInSe_2_/ZnS 4:1-H_2_O	−6.2	6.43
CuInSe_2_/ZnS 4:1-SDS	−39	7.80
